# Multi‐omics predicts radiotherapy response in small cell lung cancer patients receiving whole brain irradiation

**DOI:** 10.1002/acm2.70466

**Published:** 2026-01-22

**Authors:** Yifan Lei, Han Bai, Chengshu Gong, Yaoxiong Xia, Yu Hou, Ruiling Yang, Jinhui Yu, Zhe Zhang, Li Wang, Bo Li, Li Wang, Lan Li

**Affiliations:** ^1^ The Third Affiliated Hospital of Kunming Medical University Kunming Medical University Kunming Yunnan China; ^2^ Department of Radiation Oncology The Third Affiliated Hospital of Kunming Medical University Yunnan Cancer Hospital Kunming Yunnan China

**Keywords:** dosiomics, machine learning, radiomics, small cell lung cancer, whole brain radiotherapy

## Abstract

**Objective:**

Dosiomics and radiomics elaborate the low‐and high‐order features extracted from images to predict clinical outcomes. Whole‐brain radiotherapy (WBRT) has been widely used in patients with diffuse brain metastases of small cell lung cancer (SCLC). The objective of this study is to ascertain the predictors of treatment response in patients with SCLC treated with WBRT. Furthermore, the study seeks to develop accurate machine learning models to predict the radiotherapy response of WBRT.

**Materials and methods:**

This study retrospectively enrolled BM patients who received whole brain irradiation in Yunnan Cancer Hospital from January 2020 to June 2024. Radiomics features and dosiomics features were extracted from pre‐treatment CT images and dose images of TPS using 3D slicer software, features were screened by Least Absolute Shrinkage and Selection Operator (LASSO) regression, and Logistic Regression (LR) models assessed the association of the features with WBRT reaction. Patients who showed complete response (CR) or partial response (PR) were classified as the Radiation Response Group, while those with stable disease (SD) or progressive disease (PD) were categorized as the Radiation Non‐Response Group. A total of seven classification models were constructed, clinic factors (CFM)), radiomics features (RFM), dosiomics features (DFM), clinical factors combined with radiomics features (FM + RFM), clinical factors combined with dosiomics features (CFM + DFM), radiomics combined with dosiomics features (RFM + DFM), and the hybrid features combining clinical factors, radiomics, and dosiomics features (HFM). The HFM was our focus, evaluated the prediction performance of the model, used nomograms to visualize individualized Radiation Therapy (RT) response prediction, and prospectively collected a subset of patients for external validation set.

**Result:**

Based on univariate analysis combined with LASSO regression, three dosiomics features and four radiomics features related to the therapeutic effect were respectively selected from 851 dosiomics and radiomic features. Multivariate analysis indicated that concurrent chemoradiotherapy (CCRT), conformal boost radiotherapy (CBRT), radiomics, and dosiomics were independent predictors of the radiotherapy response of WBRT. The multicomponent model based on dosiomics, radiomics and clinical factors showed optimal predictive power in the patient cohort, with a mean AUC = 0.792 (95% CI 0.708–0.852), AUC of external validation set = 0.711 (95%CI 0.487–0.934) and the constructed nomogram charts have good clinical value.

**Conclusion:**

The integration of clinical parameters with dosiomics and radiomic features in a multi‐omics framework demonstrates enhanced predictive accuracy for assessing whole‐brain radiation therapy outcomes in small‐cell lung carcinoma. This comprehensive approach may facilitate clinical decision‐making by enabling more precise treatment customization and individualized therapeutic strategies.

## 1 BACKGROUND

Small cell lung carcinoma (SCLC), an aggressive neuroendocrine malignancy, demonstrates particularly rapid progression. Clinical observations indicate that between 10% to 20% of SCLC cases present with brain metastases (BM) at initial diagnosis. These intracranial metastases represent a significant contributor to mortality in the SCLC patient population,^1^ seriously threatening the quality of life of patients.^2^ For patients presenting with intracranial metastases, radiation therapy represents a cornerstone of clinical management. Given the distinct biological characteristics of small cell lung carcinoma, whole‐brain irradiation (WBRT) has become a standard therapeutic approach for metastatic cerebral lesions in this patient population.^3^ Current evidence suggests significant interpatient variability in radiotherapeutic outcomes for intracranial metastases, which appears to be influenced by multiple clinical determinants including histological classification, therapeutic protocols, and individual patient characteristics.^4^ Timely recognition of these predictive variables during the treatment initiation phase enables optimization of therapeutic approaches, potentially enhancing clinical outcomes for patients with intracranial metastases through more precise intervention selection. Many scholars have been seeking valuable indicators for the therapeutic effect of WBRT in patients with SCLC brain metastases. When Fan et al. constructed the predictive model, indicators such as gender, age, and extracranial metastasis were included.^5^ The results of Haiyan Zeng et al. in the study of risk factors for BM after WBRT in SCLC showed TNM staging, and Hyperfractionated radiotherapy was considered as a risk factor.^6^ It has been reported that biomarkers derived from tumor proteins and genes may also be associated with radiotherapy efficacy.^7^ However, the process of obtaining proteins and genes is often invasive and not universal. Whether these indicators can become predictive factors still requires more data to be studied. Radiomics, as an emerging quantitative analysis technology, is used in the diagnosis of various cancers by extracting information from imaging images in a high‐throughput manner and converting it into deeper feature information, thereby achieving quantitative research on lesions.^8^ Xu et al. have proved that radiomics features based on brain magnetic resonance imaging (MRI) as a single biomarker are expected to predict the local tumor response in patients with brain metastases from lung cancer after WBRT.^9^ Research by Delli Pizzi's team demonstrated the feasibility of forecasting treatment response to chemoradiotherapy in rectal cancer patients through MRI assessment,^10^ while Eaford, India et al. differentiated the tumor response assessment after neoadjuvant chemoradiotherapy for esophageal cancer based on radiomics of Computerized Tomography (CT) and Fluorodeoxyglucose Positron Emission Tomography (FDG‐PET).^11^ Although significant progress has been made in predicting therapeutic efficacy, it is still necessary to further improve the predictive performance. Similar to radiomics, the research method of dosiomics has emerged, which is different from the information provided by traditional Dose Volume Histogram (DVH) diagrams. Dosiomics describes the dose distribution from the three‐dimensional spatial distribution.^12^, ^13^ Scholars such as Salome and Patrick improved the risk stratification of high‐grade gliomas receiving carbon ion radiotherapy by integrating radiomics and dosiomics features.^14^ The radiomics and dosiomics features extracted from the pre‐processed MRI images by Zhang et al. successfully predicted the treatment response of BM patients after PULSAR treatment, with an Area Under the Curve (AUC) of 0.979.^15^This indicates that dosiomics, as a new predictor, can optimize the predictive performance of the model. Therefore, based on the clinical characteristics, this study combined dosiomics and radiomics, hoping to construct a more accurate multi‐omics response prediction model. In addition, the differences among various omics models were explored to identify the related factors affecting the radiotherapy efficacy of SCLC patients with brain metastases, so as to help optimize the treatment strategy.

## 2 MATERIALS AND METHODS

**FIGURE 1:**
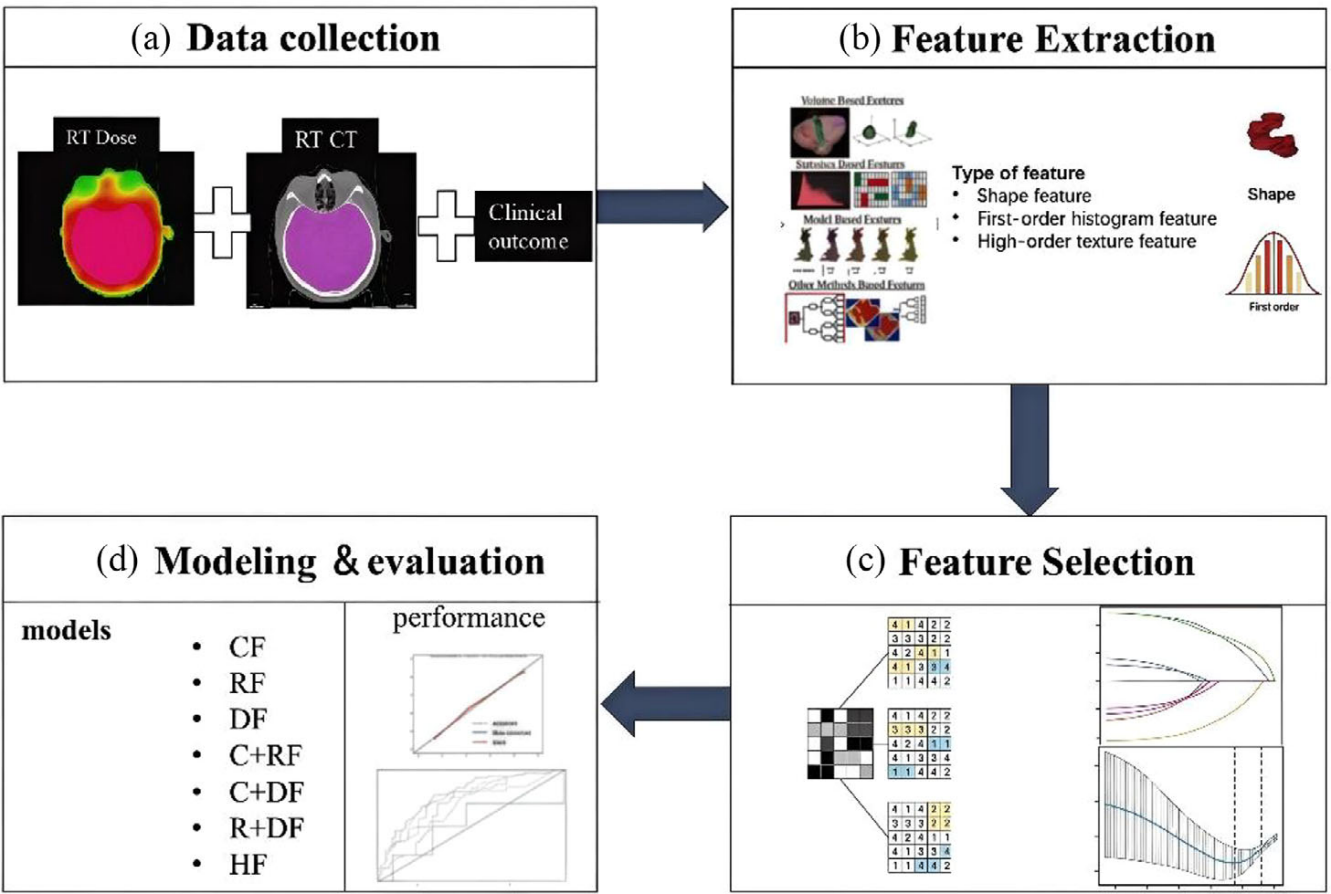
Workflow for building the predictive classifier.

The construction of the model in this study was divided into four parts (Figure 1): (a) Data collection, including clinical data, pre‐treatment cranial CT images, and radiotherapy dose images; (b) Feature extraction. Multi‐omics features were based on clinical features, preprocessed planned CT images, and 3D radiotherapy dose images, and dosiomics and radiomics features of the whole brain irradiation area (CTV) were extracted; (c) Feature selection. Considering the redundancy and correlation of features, clinical features were screened using single/multi‐factor screening, and omics features were screened using single‐factor combined with lasso regression models; (d) Modeling and evaluation. To develop the radiotherapy response classifier, we employed a logistic regression algorithm incorporating the identified predictive features. The performance of the model was evaluated and internally validated within the study cohort, and external validation at independent times was performed to further evaluate the generalization ability of the model, all preprocessing steps (scaling, resampling, feature selection) were done strictly within the training folds to prevent any data leakage. Additional methodological details are presented below.

## 3 PATIENT DATA

**FIGURE 2:**
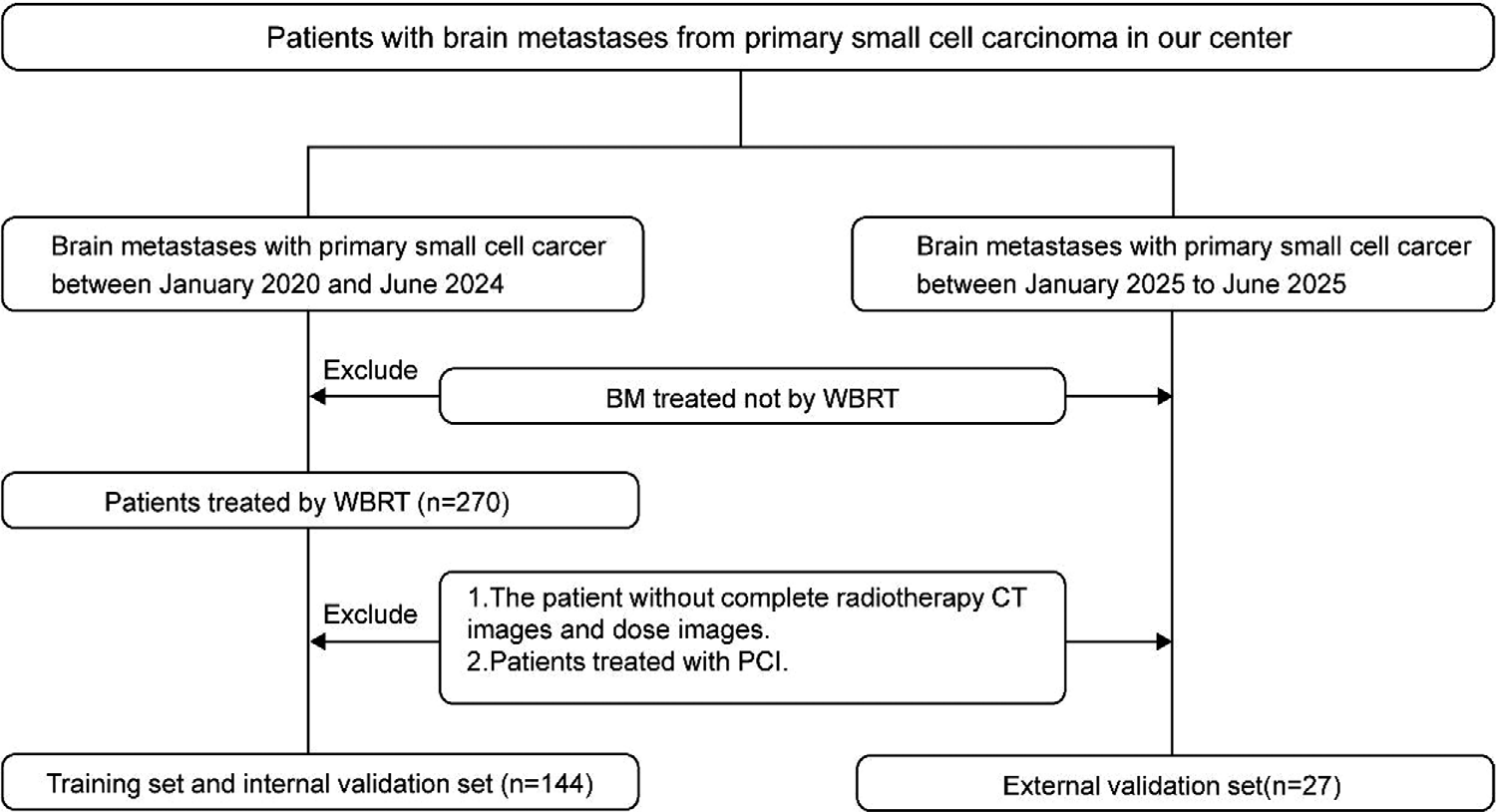
Flow chart of patient selection for this retrospective study.

This study retrospectively analyzed BM patients treated at Yunnan Cancer Hospital from January 2020 to June 2024, the data of patients from January to June 2025 were prospectively collected as an independent external validation set. The inclusion criteria were (1) BM with metastasis from primary SCLC; (2) BM treated with WBRT; (3) Complete CT images and dose images on radiotherapy TPS; (4) Age ≥ 18 and complete clinical data. The clinical factors such as age, TNM stage, brain metastasis location, and whether immunotherapy was used were collected. The screening flowchart is shown in (Figure 2). Radiation therapy was uniformly delivered via volumetric modulated arc therapy (VMAT) with 6‐MV photon beams generated by an Elekta linear accelerator. Treatment planning was performed using either Pinnacle (Philips, Netherlands) or Monaco (Elekta, Sweden) treatment planning systems, The radiotherapy plan was calculated based on the Monte Carlo algorithm, and the dose grid resolution was set to 0.3 cm, with dose prescription normalized such that 95% of the planning target volume (PTV) received the intended dose. The primary outcome of the study was the rate of short‐term tumor response to WBRT (defined as the result of the first head MRI follow‐up within 1 to 3 months). According to the RANO‐BM criteria, it was divided into the remission group: complete remission (CR) + partial remission (PR), and the non‐remission group: stable disease (SD) + progressive disease (PD). Other outcomes, such as progression‐free survival and overall survival, were also studied.

## 4 RADIOMICS AND DOSIOMICS FEATURE EXTRACTION

The current investigation incorporated two distinct omics feature categories for radiotherapy response prediction. Prior to analysis, all patient‐derived DICOM‐formatted computed tomography scans and radiation dose distribution images underwent standardized preprocessing procedures. Through 3D Slicer software ^16^ (version 5.6.2, https://www.slicer.org), the images with the contour target volume (including image and dose images) were imported for visualization. The region of interest (ROI) was segmented using the Segment Editor module. Before feature extraction, the CT images and dose images were resampled to a voxel size of 1×1×1 mm^3^, and the radiomics features were automatically extracted using the Radiomics module. The bin width for extracting features was set to 25. A total of 107 original features were extracted from the image and dose images respectively, including shape (*n* = 14), first‐order (*n* = 18), and texture (*n* = 75). Then, the original features were filtered by wavelet transform to generate additional feature sets, and a total of 851 omics features were extracted. Feature extraction parameter setting file conforming to ibsi level, it has been provided in (Dataset S‐1).

## 5 OMICS FEATURE SELECTION AND THE PRODUCTION OF OMICS SCORES

The least absolute shrinkage and selection operator (LASSO) algorithm, a well‐established statistical approach for high‐dimensional feature selection,^17^ was employed in this investigation. Feature selection was performed through a two‐stage process: preliminary screening via univariate analysis followed by application of LASSO regression to identify radiomic and dosiomics characteristics significantly associated with therapeutic response to radiation. The LASSO algorithm applies coefficient shrinkage based on the tuning parameter *λ*, driving non‐predictive features to exact zero values while retaining relevant predictors. Subsequently, the radiomics and dosiomics signatures were computed as weighted sums of the retained features, with each variable's contribution determined by its corresponding regression coefficient.

## 6 MODELING AND EVALUATION

We constructed seven classification models to predict the tumor response rate of patients after WBRT. Until the end of the follow‐up period, clinical factors (CF model (CFM)), radiomics features (RF model (RFM)), dosiomics features (DF model (DFM)), clinical factors combined with radiomics features (C + RF model (CFM + RFM)), clinical factors combined with dosiomics features (C + DF model (CFM + DFM)), radiomics combined with dosiomics features (R + DF model (RFM + DFM)), and the hybrid features combining clinical factors, radiomics, and dosiomics features (HF model (HFM)) were used. To avoid inflating type‐I errors with multiple model variants, we declare that HFM is the main model we constructed, and the others are exploratory models. The process of building all models included three steps: (1) Feature selection and modeling. Univariate analysis was used to screen the factors related to the therapeutic effect. Factors with *p* < 0.05 were included in the multivariate analysis, and the logistic regression algorithm was used to model the dataset; (2) Evaluation of model performance. Multiple evaluation metrics were used to compare the performance of the models, including the area under the ROC curve (AUC), PR‐AUC, accuracy, sensitivity, specificity, False positive rate and f1 score, clinical decision curve. For the optimal prediction model, the calibration curve was plotted. (3) Evaluation of the generalization ability of the optimal model. bootstrap internal sampling was performed 1000 times for validation, a nomogram was drawn based on feature weights for visualization, and an external validation set was used for validation.

## 7 STATISTICAL ANALYSIS

Categorical variables were analyzed using either the *χ* ^2^ test or Fisher's exact test, as appropriate. Continuous variables were evaluated with Student's *t*‐test or the Mann–Whitney U test, depending on data distribution. Survival analysis was conducted using Kaplan‐Meier curves, while predictive modeling employed multivariate logistic regression. Diagnostic performance was assessed through ROC curve and PR‐AUC analysis (SPSS v26.0, IBM). Model validation included calibration plots, decision curve analysis (DCA), and nomogram construction (R v3.0.1). A significance threshold of *p *< 0.05 was applied throughout all analyses.

## 8 RESULT

### 8.1 Patient characteristics

Following predefined selection criteria, we retrospectively analyzed 144 small cell lung carcinoma cases (male: *n* = 131, 91.0%; female: *n* = 13, 9.0%). Based on treatment response, patients were stratified into two cohorts: responders (*n* = 74) and non‐responders (*n* = 70). Because of the completeness of the clinical data, the percentage of missing data was very low (< 5%) and had little predictive power in previous studies, so we used a single imputation method (modal imputation method) for missing clinical data, this approach does not change the predictions of the model. Table 1 presents the complete demographic and clinical characteristics of the study population.

**TABLE 1:** Clinical parameters of the patient population.

Variables	Total (*n* = 144)	Response group (*n* = 74)	Non‐response group (*n* = 70)
Age, Mean ± SD	59.69 ± 8.55	59.85 ± 8.78	59.53 ± 8.35
Size of BM, Mean ± SD	1.69 ± 1.12	1.75 ± 1.1	1.62 ± 1.11
Gender, *n*(%)			
Male	131 (90.97)	70 (94.59)	61 (87.14)
Female	13 (9.03)	4 (5.41)	9 (12.86)
KPS, *n*(%)			
≤70	25 (17.36)	15 (20.27)	10 (14.29)
>70	119 (82.64)	59 (79.73)	60 (85.71)
T, *n*(%)			
1	11 (7.64)	7 (9.46)	4 (5.71)
2	35 (24.31)	16 (21.62)	19 (27.14)
3	20 (13.89)	8 (10.81)	12 (17.14)
4	78 (54.17)	43 (58.11)	35 (50.00)
N, *n*(%)			
0	7 (4.86)	3 (4.05)	4 (5.71)
1	8 (5.56)	3 (4.05)	5 (7.14)
2	51 (35.42)	24 (32.43)	27 (38.57)
3	78 (54.17)	44 (59.46)	34 (48.57)
M, *n*(%)			
0	60 (41.67)	32 (43.24)	28 (40.00)
1	84 (58.33)	42 (56.76)	42 (60.00)
Clinical Stages, *n*(%)			
Limited‐stage	65 (45.14)	35 (47.30)	30 (42.86)
Extensive‐stage	79 (54.86)	39 (52.70)	40 (57.14)
CCRT, *n*(%)			
No	87 (60.42)	37 (50.00)	50 (71.43)
Yes	57 (39.58)	37 (50.00)	20 (28.57)
NDBM, *n*(%)			
No	93 (64.58)	45 (60.81)	48 (68.57)
Yes	51 (35.42)	29 (39.19)	22 (31.43)
Chemotherapy, *n*(%)			
≤4 weeks	34 (23.61)	17 (22.97)	17 (24.29)
>4 weeks	110 (76.39)	57 (77.03)	53 (75.71)
Immunotherapy, *n*(%)			
No	112 (77.78)	52 (70.27)	60 (85.71)
Yes	32 (22.22)	22 (29.73)	10 (14.29)
LM, n(%)			
No	137 (95.14)	72 (97.30)	65 (92.86)
Yes	7 (4.86)	2 (2.70)	5 (7.14)
Location of BM, *n*(%)			
Supratentorial	73 (50.69)	38 (51.35)	35 (50.00)
Infratentorial	18 (12.50)	7 (9.46)	11 (15.71)
Supratentorial +Infratentorial	53 (36.81)	29 (39.19)	24 (34.29)
BMS, *n*(%)			
No	90 (62.50)	44 (59.46)	46 (65.71)
Yes	54 (37.50)	30 (40.54)	24 (34.29)
CBRT, n(%)			
No	93 (64.58)	41 (55.41)	52 (74.29)
Yes	51 (35.42)	33 (44.59)	18 (25.71)

Abbreviations: BMS, Brain Metastasis Symptoms; CBRT, concurrent boost; CCRT, Concurrent Chemoradiotherapy; LM, Leptomeningeal metastasis; NDBM, Newly Diagnosed Brain Metastasis; SD, standard deviation; t, *t*‐test, χ^2^, Chi‐square.

### 8.2 The generation of omics scores

**FIGURE 3:**
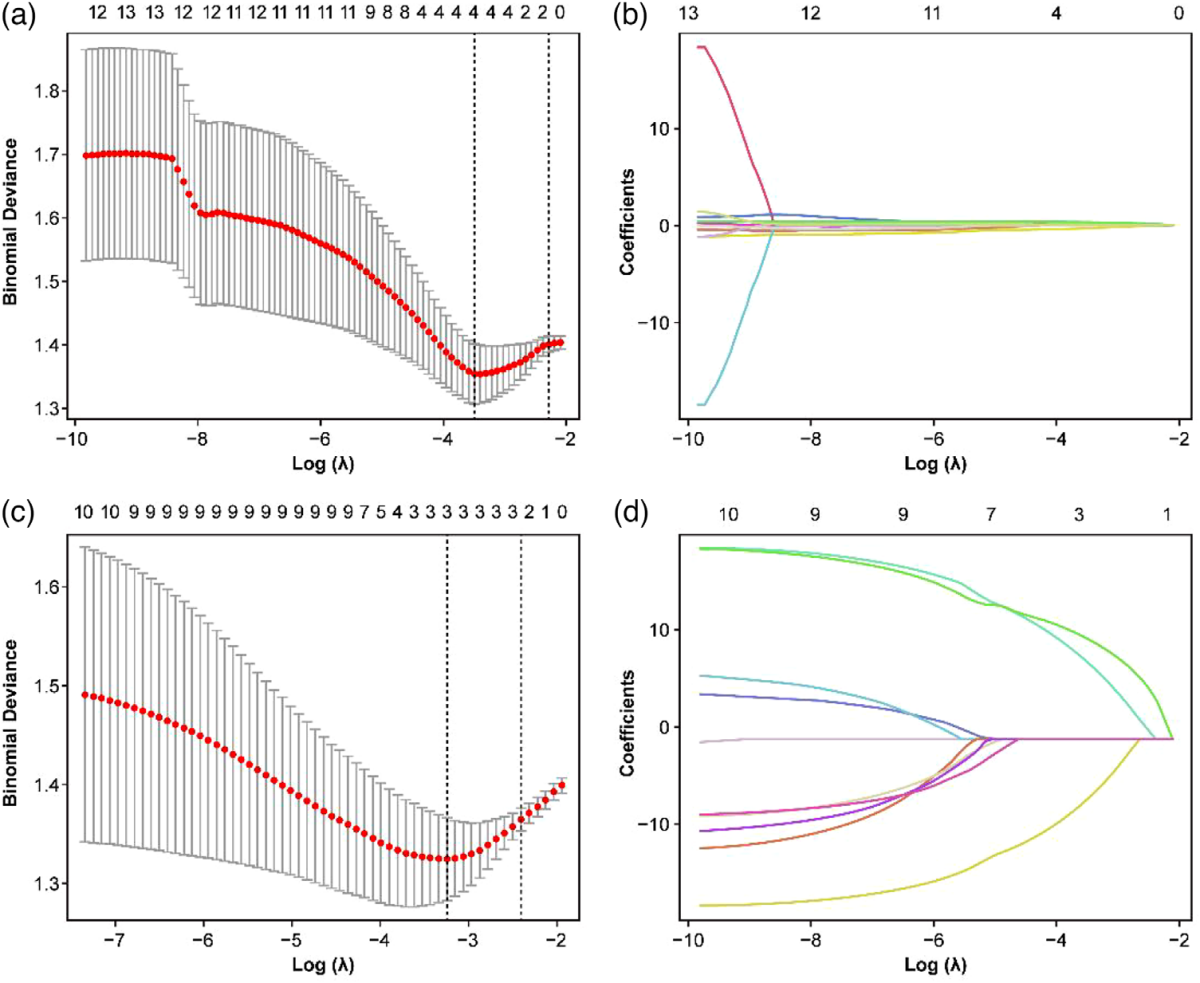
Predictive radiomic features were identified through LASSO regression analysis. (A, C) Model optimization employed 10‐fold cross‐validation, withλselection guided by maximizing ROC‐AUC while minimizing standard deviation. (B, D) Feature coefficient profiles demonstrate the relationship between dosiomics/radiomic feature weights and regularization strength (L1 norm), plotted against the negative logarithmicλscale.

It involves first conducting univariate analysis on the omics features to screen out the differential features. (As shown in Figure 3), the screened omics features were subjected to *Z*‐score standardization and then underwent tenfold cross‐validation in the elastic network to adjust the parameter λ, thereby selecting the radiomics features and dosiomics features related to the radiotherapy efficacy. According to the LASSO regression analysis, four radiomics features (original glcm, wavelet‐LLH firstorder, wavelet‐HLL glszm, wavelet‐LLL firstorder) and three dosiomics features (original shape, wavelet‐HHL glszm, wavelet‐HHH glszm) were respectively screened out from the two omics datasets, Details Feature information are provided in (Table S‐1). Individual patient omics scores were calculated using linear combinations of selected features weighted by coefficients (Dataset S‐2).

### 8.3 Model training and evaluation

**FIGURE 4:**
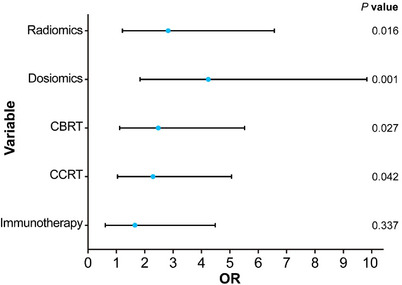
The multi‐factor forest map constructed by five factors is shown, OR:Odds Ratio.

**FIGURE 5:**
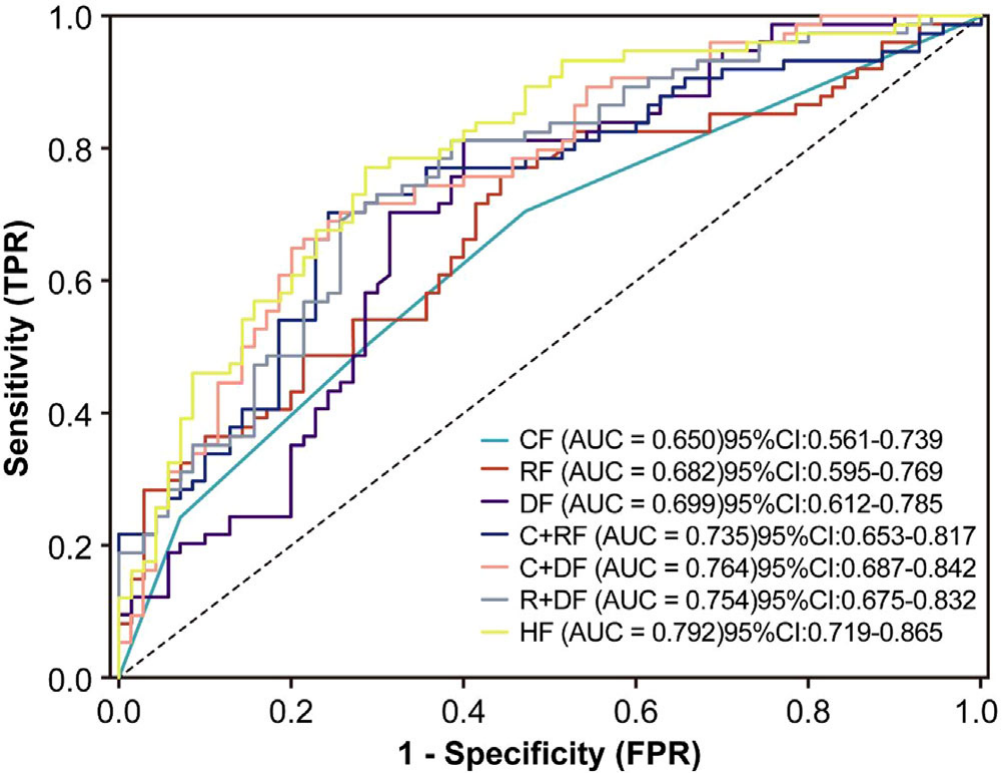
ROC curves were plotted for individual and combined feature models.

**FIGURE 6:**
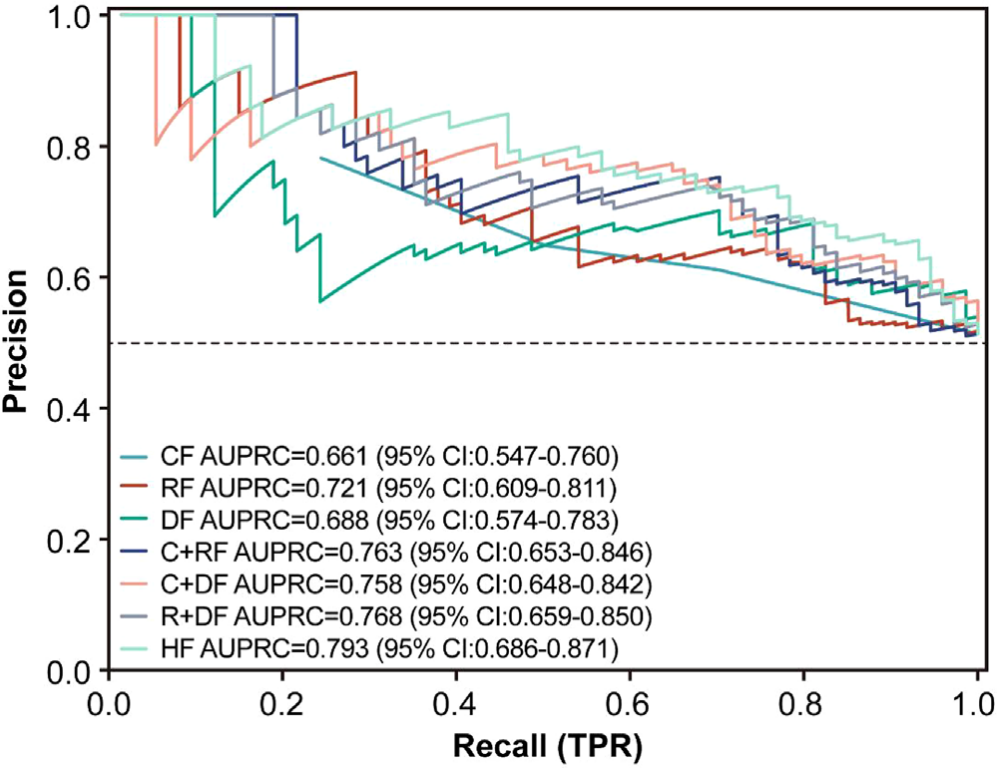
PR‐AUC curves were plotted for individual and combined feature models.

**FIGURE 7:**
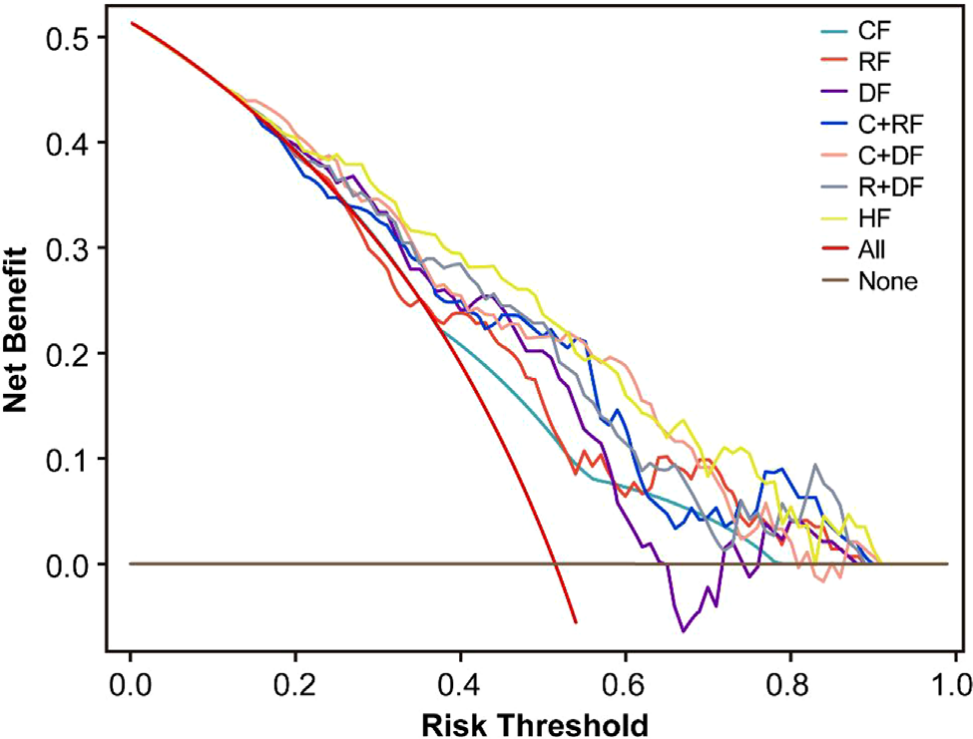
Comparison of clinical decision curves of the seven constructed models in the cohort.

**FIGURE 8:**
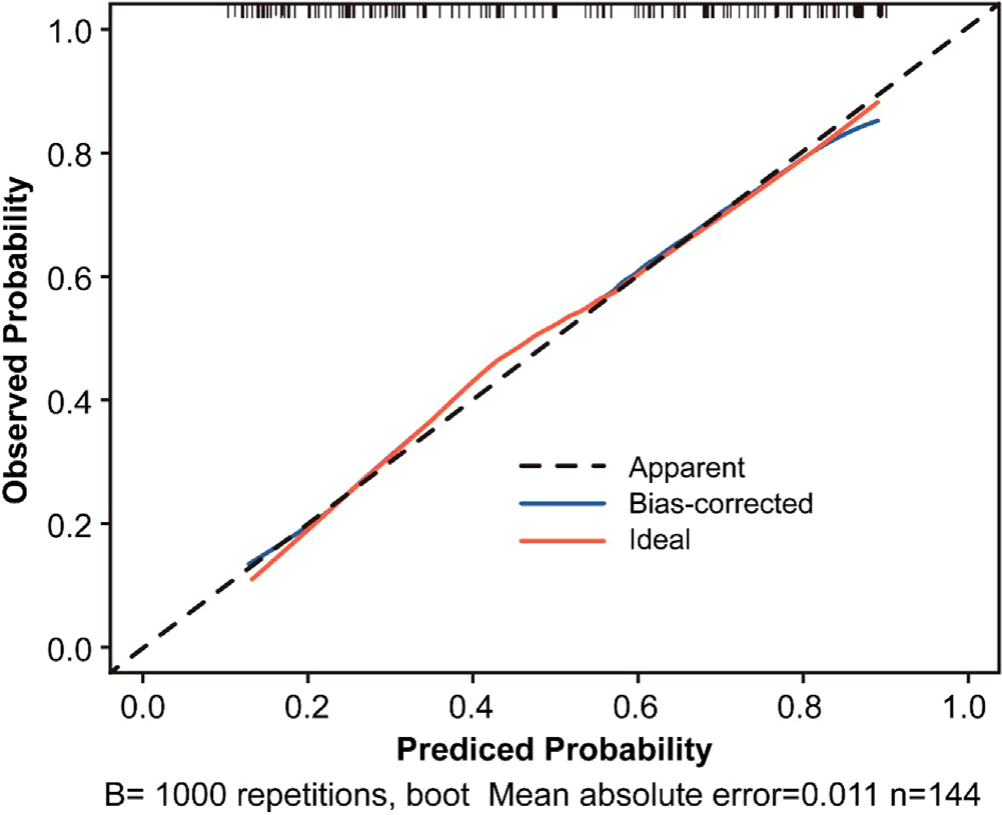
Calibration curves plotted by the optimal mixture model HF.

**FIGURE 9:**
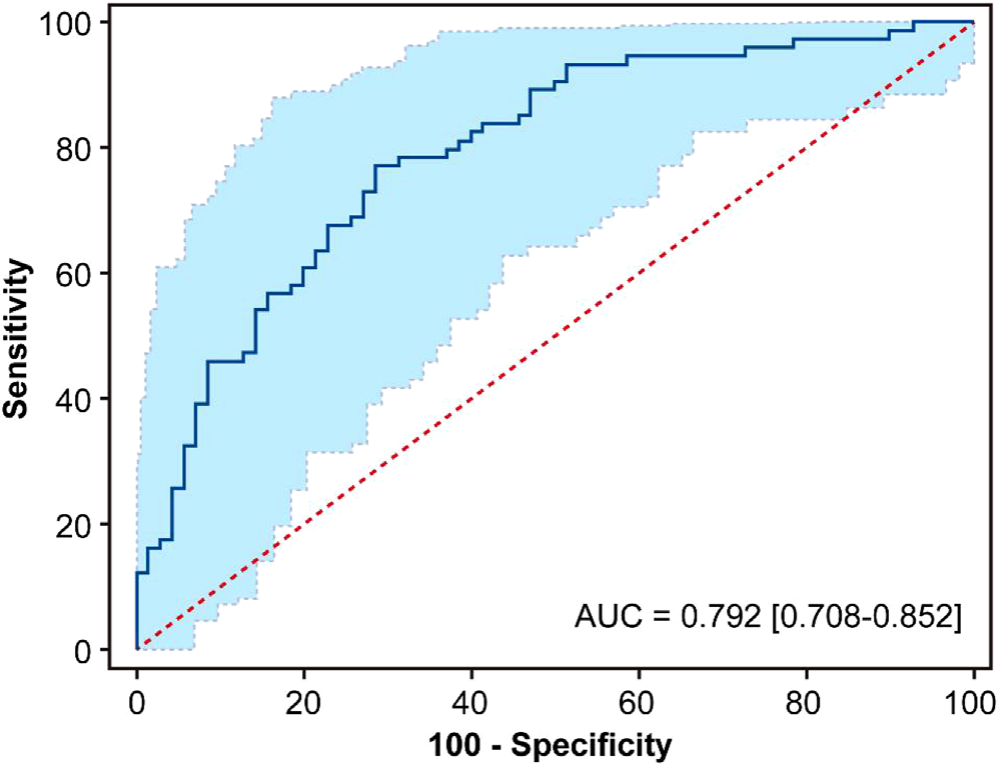
ROC plot of the HF model with 1000 bootstrap resampling for internal validation.

**FIGURE 10:**
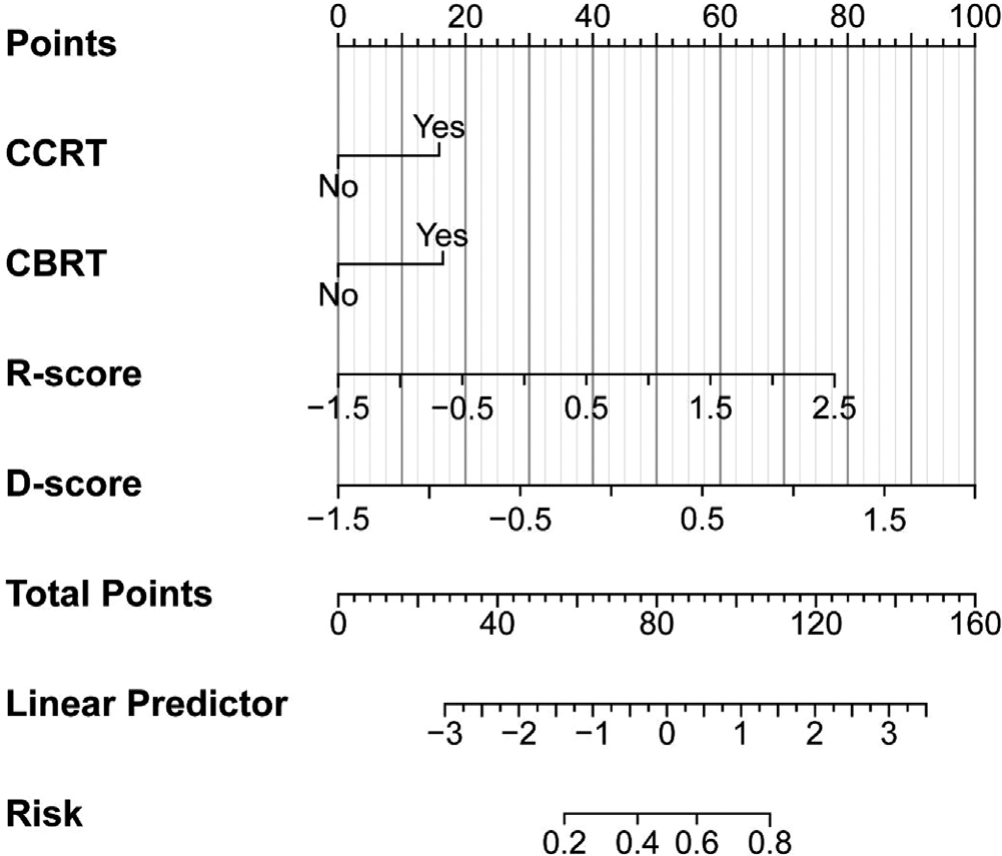
Nomogram based on weight of clinical factors, radiomics features and dosiomics features was constructed.

**FIGURE 11:**
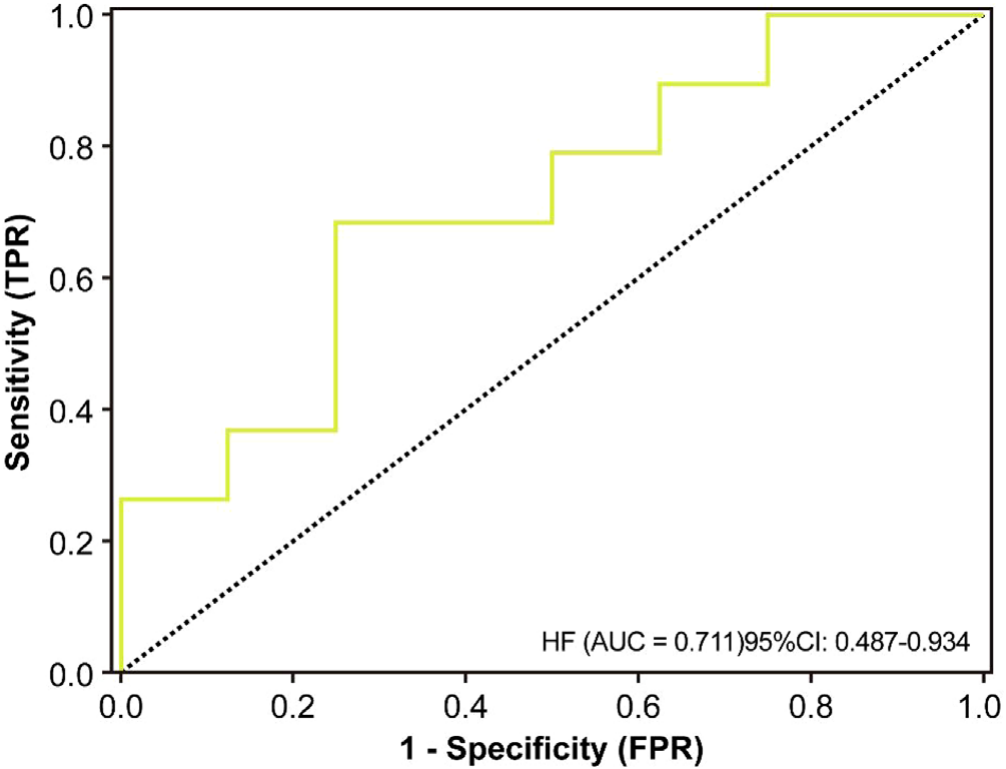
The ROC curve of the nomogram was plotted in the external validation cohort.

Table 2 presents the univariate and multivariate logistic regression analyses identifying significant predictors of radiotherapeutic outcomes in the study population. (Figure 4) presents the forest plot of the multivariate analysis. Here, immunotherapy was excluded. According to the multivariate analysis, concurrent chemoradiotherapy, local radiotherapy dose escalation, Dosiomics Score and Radiomics Score are independent predictors of the therapeutic effect of radiotherapy. Seven classification models were constructed based on the analysis results using the Logistic classification algorithm. We noted model stability and performed a quantitative evaluation; events Per Variable (EPVs) is shown in (Table S‐2). Table 3 shows the prediction results of different comparison models. We observed that dosiomics combined with clinical features and dosiomics combined with radiomics had similar predictive performances (i.e., C + DF and R + DF). (Figure 5) presents the comparison of the ROC curves of the models, and the hybrid model (HF) has the best predictive ability, the model performed well in the clinical key interval where the false positive rate was ≤10%, and the partial AUC reached 0.038, suggesting that it has important application potential in clinical decision scenarios requiring high specificity. (figure 6) shows the comparison of PR‐AUC curves of the models, in which the mixed model (HF) also has the strongest ability to find positive samples under different thresholds. (Figure 7) Decision curve analysis (DCA) was used to evaluate the clinical benefit of the constructed model. Within the threshold range of 10% to 90%, combined with the false positive rate of 0.286 and DCA curve comparison, the results show that the HF model has practical application value in actual clinical application, and the net benefit is the highest. (Figure 8) The calibration curve of the optimal model HF showed that the Brier score was 0.011, the slope was 1.02, and the intercept was ‐0.031. The calibration and discrimination of the model were good. This study was a single‐center study. To make full use of the data and improve the robustness of the results, the Bootstrap method (1000 times) was used for internal validation of the data. The results are shown in (Figure 9), with an average AUC of 0.792 [95% CI (0.708–0.852)], cut‐off value 0.483, Youden index 0.485, sensitivity 0.770, specificity 0.714, and the model fit was good. A nomogram (Figure 10) was constructed by integrating dosiomics features, radiomics features, and clinical factors to predict the radiotherapy efficacy of WBRT patients. In order to further verify the generalization ability of the nomogram, we prospectively collected a part of patients as an external validation set. The clinical baseline data of the prospectively collected patient cohort are shown in (Table S‐3). (Figure 11) shows the prediction performance of the nomogram in the external validation set. The ROC curve showed AUC = 0.711,95%CI:0.487–0.934, The model had good generalization ability.

**TABLE 2:** Predictors of radiotherapeutic response: Univariate and multivariate logistic regression analyses.

	Univariate analysis			Multivariable analysis			
Variable	*p* value	Variable	*p* value	Variable	OR	95%CI	*p* value
Gender	0.119	NDBM	0.33	Immunotherapy	1.638	0.598∼ 4.484	0.337
Age	0.822	Chemotherapy	0.853	CCRT	2.282	1.028 ∼ 5.064	0.042^*^
MD	0.487	Immunotherapy	0.026^*^	CBRT	2.476	1.110 ∼ 5.524	0.027^*^
KPS	0.343	Location of BM	0.503	D‐score	4.24	1.823 ∼ 9.859	0.001^*^
T	0.46	LM	0.216	R‐score	2.822	1.212 ∼ 6.573	0.016^*^
N	0.574	BMS	0.438				
M	0.693	CBRT	0.018^*^				
Clinical Stages	0.593	CCRT	0.009^*^				
R‐score	<0.001^*^	D‐score	<0.001^*^				

Abbreviations: D‐score, Dosiomics score; R‐score, Radiomics score

* Indicates a statistical difference.

**TABLE 3:** Comparison of the prediction performance of the seven constructed models.

model	AUC	ACC	SEN	SPE	FPR	F1‐score
CF	0.65	0.618	0.703	0.529	0.471	0.615
RF	0.682	0.639	0.757	0.557	0.443	0.638
DF	0.699	0.694	0.811	0.600	0.4	0.694
C+RF	0.735	0.701	0.703	0.757	0.243	0.701
C+DF	0.764	0.701	0.649	0.800	0.2	0.701
R+DF	0.754	0.715	0.689	0.743	0.257	0.715
HF	0.792	0.722	0.770	0.714	0.286	0.722

Abbreviations: ACC, Accuracy, AUC, Area Under the Curve; FPR, False Positive Rate; SEN, Sensitivity; SPE, Specificity

## 9 DISCUSSION

Patients with SCLC have a high risk of intracranial diffuse metastatic disease, and WBRT remains the first‐line treatment for SCLC patients with brain metastases.^18^ The era of precision medicine has revealed substantial intratumoral heterogeneity across genomic profiles, molecular pathways, and phenotypic characteristics, with significant implications for patient outcomes.^19^ This is also significant among different regions of the same lesion in patients, such as metastatic tumors,^20^ leading to different responses or resistances to radiotherapy or chemotherapy. Therefore, clinical features may not fully capture the heterogeneity of the tumor, and it is usually difficult to predict the efficacy of BM. Quantitative radiomic and dosiomics analyses enable the extraction of subclinical imaging biomarkers, offering potential solutions to current limitations in tumor characterization. The integration of machine learning algorithms with these omics approaches shows particular promise for advancing precision oncology and personalized therapeutic strategies,^21^ allowing multiple factors to be considered in a multidimensional model. Research by Fei's team validated the predictive value of radiomic features for SBRT outcomes in NSCLC patients.^22^ Using SVM‐based learning, the classifier attained 74.8% predictive accuracy in model training. Chu et al. used radiomics of primary tumors and pre‐metastatic brain features to predict NSCLC patients who are more likely to have BM,^23^ and both achieved gratifying results. Secondly, most of the dosiomics predictors included in the construction of the prediction model currently use DVH parameters, which cannot well reflect the spatial specificity between the irradiation dose and the target area. Dosiomics shows great advantages in this aspect. Therefore, this study combines radiomics and dosiomics to study the efficacy of whole‐brain irradiation in SCLC and construct a prediction model.

**FIGURE 12:**
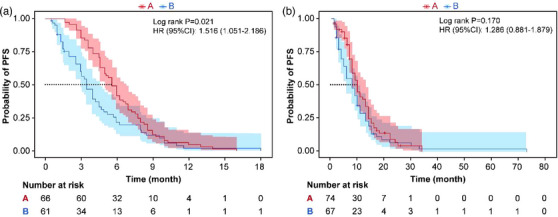
Kaplan‐Meier survival curves were generated according to the group of patients in cohort a with response and cohort b without response. A is the PFS curve and B is the OS curve.

In this study, we evaluated the post‐radiotherapy efficacy of SCLC patients who received WBRT using VMAT radiotherapy technology. We used several models of single omics (CF, RF, DF) and established a multi‐omics model to predict the objective response rate of tumor patients. The model performance demonstrated the feasibility of jointly preprocessing CT images and dose images to predict the radiotherapy efficacy, which is similar to the results obtained by Salome, Patrick, et al. using a multimodal model of radiomics and dosiomics features (RD).^14^ The predictive performance of the multi‐omics feature model is much greater than that of the single‐omics feature. The AUC of the HF model is 0.792, which was also consistent with the research results of xu using MRI images to predict local response after whole brain irradiation.^9^ Buizza et al. also achieved a C‐index of 0.8 in the prediction performance of the multi‐omics model of radiomics combined with dosiomics in their study of local control of skull base chordoma after radiotherapy.^24^ We also found that the selected features all belong to spatial texture features. The radiomics texture features may reflect the radiosensitivity of brain tissue affecting the tumor efficacy, which has also been mentioned in previous studies.^9^ In this study, the predictive ability of the RF model reached an AUC of 0.682, which was similar to the results of the nomogram constructed by integrating clinical features and radiomics features proposed by Zhang et al., among which the AUC of the individual radiomics model reached 0.635. The key factor that makes dosiomics crucial for evaluating the efficacy of tumor radiotherapy may be that during the planning optimization, even though the target volume of each patient reaches 95%, the location of the underdose in the planned target area is different, which is often the key to the non‐remission of the tumor after radiotherapy for patients. It is worth noting that the predictive ability of the DF model is stronger than that of the RF model, indicating that compared with radiomics, dosiomics features have more obvious advantages in predicting the efficacy of tumors in whole‐brain irradiation. This result is inconsistent with previous studies. In this study, showed superior predictive accuracy of the RF classifier over the DF model in anticipating radiotherapy‐related esophageal toxicity, and no dosiomics feature was retained in the construction of the hybrid model. The possible reasons for this result may be as follows: (1) All the patients enrolled in this study received WBRT treatment, and the extracted feature ROI area was the whole brain CTV. The radiomics information had a high overlap with the information under dose coverage. We conducted Pearson correlation test on the two sets of features, and the correlation coefficient was 0.236 (*p* = 0.004), indicating a relatively high correlation. The excessive repetition of the two sets of features led to the effect of radiomics features being masked. (2) Dosiomics can reflect the subtle differences in dose, and this difference may be more manifested in the radiotherapy efficacy. In addition, we also investigated clinical factors. Previous studies related to dosiomics have included patients with different clinical backgrounds, such as different clinical stages, pathology, chemotherapy regimens, and target dose.^25^ Similar to our research results, the predictive ability of clinical factors for radiotherapy efficacy was poor, with CFAUC = 0.65 (*p* = 0.003). To avoid confounding of the results by treatment route, Analyses of treatment patterns were performed according to prespecified subgroups, and the results are presented in (Figure S‐1, S‐2, S‐3). CCRT and CBRT as independent predictive factors have been reported in previous studies. The study by Rades, D et al. found that WBRT combined with CBRT could significantly improve local control of metastases.^18^ The sensitizing effect of concurrent chemoradiotherapy on radiotherapy has been extensively studied.^27^, ^28^ The controlled clinical investigation demonstrated significantly improved overall survival (OS) in SCLC patients with cerebral metastases when treated with combined whole‐brain radiotherapy and chemotherapy, whereas neither therapeutic modality alone conferred significant survival advantages.^29^ The results of a meta‐analysis of different treatment options for brain metastases from non‐small cell lung cancer show that concurrent chemoradiotherapy enhances the efficacy of metastatic disease.^30^ It is worth noting that the analysis of PFS and OS survival curves revealed that patients in the Radiation Response Group tended to have a longer progression‐free survival period, but there was no significant difference in OS between the two groups (Figure 12).

However, this conclusion is limited by the limited number of enrolled patients. The relationship between radiotherapy efficacy and prognosis in patients with small cell brain metastases is still controversial, and further research is necessary in the future. Several methodological constraints should be acknowledged in this investigation. First, the retrospective nature of the analysis may introduce potential biases. Second, while the predictive nomogram demonstrated satisfactory performance during internal validation using institutional data, its generalizability requires confirmation through external validation with independent multicenter cohorts. thirdly test‐retest or ICC‐based stability analysis was not performed in this study and could be explored in future work. It should also be noted that some different segmentation schemes were used in the study, which may lead to different tumor responses, and different radiotherapy accelerators may cause instability of dosiomics features. Previous studies have shown that grid resolution and algorithmic dose calculation may affect certain dosiomics features.^13^ Finally, this study did not include WBRT patients with non‐SCLC, such as breast cancer, NSCLC, etc. In the subsequent studies, we are prepared to share patient data across different datasets and draw a more comprehensive nomogram, which is expected to improve the ability to predict the radiotherapy efficacy of patients and generate more universal models in the future. The TRIPOD‐AI report for this study is provided in (Table S‐4) and Study codes are available in (Dataset S‐3).

## 10 CONCLUSION

In conclusion, this study provides the concept that dosiomics features can be used as a novel biomarker to predict treatment response in patients receiving WBRT. The nomogram based on clinical factors, radiomics features of CT images and dosiomics features of radiotherapy images is associated with the radiotherapy efficacy of SCLC patients with brain metastases receiving WBRT, which is helpful for adjusting and selecting the correct treatment strategy, thereby improving the prognosis of these patients.

## AUTHOR CONTRIBUTIONS

Yifan Lei, Han Bai and Chengshu Gong wrote the first draft of the manuscript and are co‐first authors. Lan Li and Li Wang conducted the study and critically revised the manuscript. Yaoxiong Xia and Yu Hou analyzed the data. Chengshu Gong and Ruiling Yang collected the data, and Zhe Zhang and Jinhui Yu Data and pictures were processed. Li Wang and Bo Li prepared tables for the article. All authors contributed to the manuscript and approved the version as submitted.

## CONFLICT OF INTEREST STATEMENT

This paper, totally or partly, has not been published or submitted elsewhere. There is no conflict of interest in whichever form at the submission of this manuscript.

## ETHICAL APPROVAL

This study has been approved by the Ethics Committee of Yunnan Cancer Hospital, and the project ethics number is KYLX2024‐028.

## STATEMENT OF APPRECIATION

Thanks to all the people at Yunnan Cancer Hospital who contributed to this article.

## References

[1] Siegel RL , Giaquinto AN , Jemal A . Cancer statistics, 2024. CA‐Cancer J Clin. 2024;74(1):12‐49. doi:10.3322/caac.21820 38230766

[2] Fariña H , Rodríguez‐Salazar M , Rodríguez‐Yanes M , et al. P14.40 Incidence and risk factors identification for lung cancer brain metastasis. Neuro‐Oncology. 2019;21(Supple 3):iii76‐iii76. doi:10.1093/neuonc/noz126.275

[3] Rusthoven CG , Yamamoto M , Bernhardt D , et al. Evaluation of first‐line radiosurgery vs whole‐brain radiotherapy for small cell lung cancer brain metastases: the FIRE‐SCLC Cohort Study. JAMA Oncol. 2020;6(7):1028‐1037. doi:10.1001/jamaoncol.2020.1271 32496550 PMC7273318

[4] Baltalarli B , Yalman D , Akagündüz O , et al. Identification of prognostic factors in patients with brain metastases: a review of 493 patients. J Clin Oncol. 2006;24(18_suppl):11516‐11516. doi:10.1200/jco.2006.24.18_suppl.11516

[5] Fan Z , Huang Z , Tong Y , et al. Sites of synchronous distant metastases, prognosis, and nomogram for small cell lung cancer patients with bone metastasis: a Large Cohort Retrospective Study. J Oncol. 2021:9949714. doi:10.1155/2021/9949714 34367286 PMC8342177

[6] Zeng H , Xie P , Meng X , et al. Risk factors for brain metastases after prophylactic cranial irradiation in small cell lung cancer. J Clin Oncol. 2016; 34 (15_suppl): e20095‐e20095. doi:10.1200/jco.2016.34.15_suppl.e20095 PMC5311871 28202905

[7] Lee, JM , Kohn, EC . Proteomics as a guiding tool for more effective personalized therapy. Ann Oncol. 2010; 21 (Suppl 7): vii205‐10. doi:10.1093/annonc/mdq375 20943616 PMC3018888

[8] Zheng, Z , Wang, J , Tan, W , et al. 18F‐FDG PET/CT radiomics predicts brain metastasis in I‐IIIA resected non‐small cell lung cancer. Eur J Radiol. 2023; 165: 110933. doi:10.1016/j.ejrad.2023.110933 37406583

[9] Xu R , Tian Y , Zhang B . MRI‐based radiomics signature for the prediction of response of lung cancer brain metastases after whole‐brain radiotherapy. J Clin Oncol. 2021; 39 (15_suppl): e21096. doi:10.1200/jco.2021.39.15_suppl.e21096

[10] Delli Pizzi A , Chiarelli AM , Chiacchiaretta P , et al. MRI‐based clinical‐radiomics model predicts tumor response before treatment in locally advanced rectal cancer. Sci Rep. 2021; 11 (1): 5379. doi:10.1038/s41598-021-84816-3 33686147 PMC7940398

[11] Eaford, I , Rishi, A , Zhang, G , et al. Association of radiomics and outcome in esophageal cancer following neoadjuvant chemo‐radiation. J Clin Oncol. 2018; 36 (4_suppl): 146. doi:10.1200/jco.2018.36.4_suppl.146

[12] Placidi, L , Cusumano, D , Lenkowicz, J , et al. On dose cube pixel spacing pre‐processing for features extraction stability in dosiomic studies. Phys Medica. 2021; 90 108‐114. doi:10.1016/j.ejmp.2021.09.010 34600351

[13] Placidi, L , Gioscio, E , Garibaldi, C , et al. A multicentre evaluation of dosiomics features reproducibility, stability and sensitivity. Cancers (Basel). 2021; 13 (15): doi:10.3390/cancers13153835 PMC8345157 34359737

[14] Salome, P , Sforazzini, F , Kudak, A , et al. Improved risk stratification via integration of radiomics and dosiomics features in patients with recurrent high‐grade glioma undergoing carbon ion radiotherapy (CIRT). J Clin Oncol. 2021; 39 (15_suppl): 2043. doi:10.1200/jco.2021.39.15_suppl.2043

[15] Zhang, H , Dohopolski, M , Stojadinovic, S , et al. Multiomics‐based outcome prediction in personalized ultra‐fractionated stereotactic adaptive radiotherapy (PULSAR). Cancers (Basel). 2023; 16 (19): 3425. doi:10.3390/cancers16193425 PMC11475788 39410044

[16] Fedorov A , Beichel R , Kalpathy‐Cramer J , et al. 3D Slicer as an image computing platform for the Quantitative Imaging Network. Magn Reson Imaging. 2012; 30 (9): 1323‐1341. doi:10.1016/j.mri.2012.05.001 22770690 PMC3466397

[17] Butler, B , Tayiab, N , Phu, S , et al. A machine learning tool to predict mortality risk among patients with metastatic cancer in outpatient oncology care. J Clin Oncol. 2021; 39 (15_suppl): 1560‐1560. doi:10.1200/jco.2021.39.15_suppl.1560

[18] Gaebe K , Li AY , Park A , et al. Stereotactic radiosurgery versus whole brain radiotherapy in patients with intracranial metastatic disease and small‐cell lung cancer: a systematic review and meta‐analysis. Lancet Oncol. 2022; 23 (7): 931‐939. doi:10.1016/S1470-2045(22)00271-6 35644163

[19] Dagogo‐Jack, I , Shaw AT . Tumour heterogeneity and resistance to cancer therapies. Nat Rev Clin Oncol. 2017; 15 (2): 81‐94. doi:10.1038/nrclinonc.2017.166 29115304

[20] Nicolson, GL . Generation of phenotypic diversity and progression in metastatic tumor cells. Cancer Metast Rev. 1984; 3 (1): 25‐42. doi:10.1007/BF00047691 6370418

[21] Yip M , Salcudean S , Goldberg K , et al. Artificial intelligence meets medical robotics. Science. 2023; 381 (6654): 141‐146. doi:10.1126/science.adj3312 37440630

[22] Cheung, BMF , Lau, KS , Lee, VHF , et al. Computed tomography‐based radiomic model predicts radiological response following stereotactic body radiation therapy in early‐stage non‐small‐cell lung cancer and pulmonary oligo‐metastases. Radiat Oncol J. 2021; 39 (4): 254‐264. doi:10.3857/roj.2021.00311 34986546 PMC8743458

[23] Chu, Xiao , Gong, Jing , Yang, Xi , et al. A “Seed‐and‐Soil” radiomics model predicts brain metastasis development in lung cancer: implications for risk‐stratified prophylactic cranial irradiation. Cancers (Basel). 2023; 15 (1): 307. doi:10.3390/cancers15010307 36612303 PMC9818608

[24] Buizza, G , Paganelli, C , D'Ippolito, E , et al. Radiomics and dosiomics for predicting local control after carbon‐ion radiotherapy in skull‐base chordoma. Cancers (Basel). 2021; 13 (2): 339. doi:10.3390/cancers13020339 33477723 PMC7832399

[25] Zheng, X , Guo, W , Wang, Y , et al. Multi‐omics to predict acute radiation esophagitis in patients with lung cancer treated with intensity‐modulated radiation therapy. Eur J Med Res. 2023; 28 (1): 126. doi:10.1186/s40001-023-01041-6 36935504 PMC10024847

[26] Lee, S , Geng, H , Zhong, H , et al. Intratumoral radiomics and dosiomics biomarkers for predicting overall survival in the RTOG 0617 Clinical Trial Int J Radiat Oncol. 2020; 108 (3): S164. doi:10.1016/j.ijrobp.2020.07.931

[27] Tang LL , Guo R , Zhang N , et al. Effect of radiotherapy alone vs radiotherapy with concurrent chemoradiotherapy on survival without disease relapse in patients with low‐risk nasopharyngeal carcinoma: a randomized clinical trial. JAMA‐J Am Med Assoc. 2022; 328 (8): 728‐736. doi:10.1001/jama.2022.13997 PMC9399866 35997729

[28] Liu X , Zhang Y , Yang KY , et al. Induction‐concurrent chemoradiotherapy with or without sintilimab in patients with locoregionally advanced nasopharyngeal carcinoma in China (CONTINUUM): a multicentre, open‐label, parallel‐group, randomised, controlled, phase 3 trial. Lancet. 2023; 403 (10445): 2720‐2731. doi:10.1016/S0140-6736(24)00594-4 38824941

[29] Li, H , Xue, R , Yang, X , et al. Best supportive care versus whole‐brain irradiation, chemotherapy alone, or wbrt plus chemotherapy in patients with brain metastases from small‐cell lung cancer: a case‐controlled analysis. Front Oncol. 2021; 11: 568568. doi:10.3389/fonc.2021.568568 33732638 PMC7957068

[30] Chen M , Wei L , Wang Q , et al. Efficacy of different therapies for brain metastases of non‐small cell lung cancer: a systematic review and meta‐analysis. Transl Lung Cancer R. 2023; 12 (4): 689‐706. doi:10.21037/tlcr-22-515 PMC10183403 37197616

